# Unraveling the Secrets Behind the Multidrug-Resistant Tuberculosis Treatment Outcome in Chronic Renal Failure Patients Requiring Hemodialysis: A Systematic Review

**DOI:** 10.7759/cureus.36833

**Published:** 2023-03-28

**Authors:** Grethel N Hernandez, Kofi Seffah, Mustafa Abrar Zaman, Nimra Awais, Travis Satnarine, Ayesha Haq, Deepkumar Patel, Sai Dheeraj Gutlapalli, Areeg Ahmed, Safeera Khan

**Affiliations:** 1 Internal Medicine, California Institute of Behavioral Neurosciences & Psychology, Fairfield, USA; 2 Internal Medicine, Piedmont Athens Regional, Athens, USA; 3 Internal Medicine, St. George's University School of Medicine, Newcastle upon Tyne, GBR; 4 Research, California Institute of Behavioral Neurosciences & Psychology, Fairfield, USA; 5 Pediatrics, California Institute of Behavioral Neurosciences & Psychology, Fairfield, USA; 6 Neurology, California Institute of Behavioral Neurosciences & Psychology, Fairfield, USA; 7 Internal Medicine, Richmond University Medical Center, Staten Island, USA; 8 Internal Medicine Clinical Research, California Institute of Behavioral Neurosciences & Psychology, Fairfield, USA

**Keywords:** chronic kidney disease, multidrug-resistant pulmonary tb, mycobacterium tuberculosis, pulmonary tuberculosis, renal failure, treatment outcome

## Abstract

Multidrug-resistant/rifampicin-resistant tuberculosis (MDR/RR TB) is a global concern, with 450,000 new cases and 191,000 deaths in 2021. TB and chronic kidney disease (CKD) have been associated since 1974, with suggested explanations such as oxidative stress, malnutrition, dysfunction in vitamin D metabolism, and a compromised cell-mediated immune response. End-stage renal failure patients are more likely to acquire drug resistance due to poor adherence, adverse drug reactions, and inappropriate dose adjustment. We then aim to evaluate the therapeutic outcome of multidrug-resistant TB of the lungs in patients who require hemodialysis in terms of successful treatment (cured and treatment completed) and the associated factors for a favorable outcome. Our secondary goal is to identify unfavorable treatment outcomes (treatment failed, patient died, or patient lost to follow-up) and the underlying associated factors. We conformed to the Preferred Reporting Items for Systematic Reviews and Meta-Analyses (PRISMA) 2020 Guidelines for this systematic review. We included adults (>19 years old) with chronic kidney disease who needed hemodialysis and had microbiologically confirmed multidrug-resistant pulmonary TB, excluding patients who had a renal allograft transplant, were on peritoneal dialysis, had extrapulmonary TB, were children and pregnant patients. We searched PubMed, MEDLINE, PubMed Central, ScienceDirect, Public Library of Science (PLOS), and Google Scholar. Keywords were combined with the Boolean "AND" operator to gather results as well as the medical subject heading (MeSH) search strategy. After screening study articles by reading their titles and abstracts, the following tools were used to assess the risk of bias: the Newcastle-Ottawa scale for observational studies, the Assessment of Multiple Systematic Reviews (AMSTAR) checklist for systematic reviews, and the Joanna Briggs Institute (JBI) assessment tool for case reports. Primary and secondary outcomes were assessed, and a conclusion was made.

We gathered 21,570 studies from the databases between 2013 and 2023, with 30,062 total participants. There were eight eligible studies for review. Patients with CKD, particularly those on dialysis, are at increased risk of TB due to a combination of factors that contribute to immunosuppression. TB reactivation is common in chronic renal failure patients. Diagnostic samples such as sputum and pleural fluid had lower sensitivity rates compared to tissue samples, which led to delays in diagnosis and treatment and, most importantly, contributed to drug resistance. All new dialysis patients should undergo interferon-gamma release assay testing. TB-infected patients with severe renal disease (eGFR 30 ml/min) had increased morbidity and mortality; however, the use of directly observed treatment, short-course (DOTS), and renal-dose adjustment of anti-TB medications significantly reduced these risks. Drug-induced hepatitis and cutaneous reactions were common adverse effects of anti-TB medications. A therapeutic drug monitoring guideline is required to reduce these adverse events and even mortality. Additional research is required to assess the safety and efficacy of therapeutic regimens, as well as their outcomes, in this population with multidrug-resistant TB.

## Introduction and background

Tuberculosis (TB) is an ancient illness that has not yet been eradicated. In 2021, a projected 10.6 million people were infected with TB, a 4.5% increase over 2020's figure of 10.1 million [1]. Due to the increasing number of cases, multidrug-resistant TB remains a worldwide problem. The World Health Organization (WHO) Global Tuberculosis Report 2022 identified an estimated 450, 000 new cases of multidrug-resistant/rifampicin-resistant tuberculosis (MDR/RR TB) in 2021, hence escalating the burden of drug-resistant TB. Deaths from MDR/RR-TB are estimated to reach 191,000 in 2021. On the other hand, there has been progress in the success rate for MDR/RR TB treatment, with a global success rate of 60% in 2019 (the most recent patient cohort for which data are available), demonstrating steady improvements since it was 50% in 2012.

The connection between TB and chronic renal failure has been known since 1974, with suggested explanations such as oxidative stress, malnutrition, dysfunction in vitamin D metabolism, and a compromised cell-mediated immune response [2]. Due to inflammation and toxic uremia negatively affecting immune function, end-stage renal failure patients have a heightened risk of acquiring infections, including TB [3]. These patients, primarily those on dialysis, have a 7 to 50 times more likely latent TB infection reactivating into active TB [3,4].

Patients with end-stage renal disease (ESRD) have a 92-day average delay in TB treatment due to their multitude of clinical presentations and radiographic findings [5]. During this period, ESRD patients at risk are exposed to untreated pulmonary TB patients through the nuclei of infectious droplets in hemodialysis (HD) facilities. In addition, healthcare workers promote the transmission since they have been shown to have a growing incidence of TB, especially in TB-burdened countries.

In addition, inappropriate anti-TB drug dosage leads to treatment failure or increased adverse effects [6]. Dosages are modified depending on the patient's kidney function and body weight; no change is required in mild renal failure. Saito et al. speculated how guideline-recommended kidney function-based dosage modification influences efficacy outcomes and the frequency of drug-related adverse effects in TB-infected renal failure patients [6].

Management of multidrug-resistant TB is more intricate and requires second-line drugs with more adverse effects and an extended treatment period [7]. Recommendations for the new guideline developed better outcomes under patient values and preferences. According to the previous WHO recommendation, there were at least five drugs in the intensive treatment phase, characterized primarily by the utility of second-line injectable agents. The American Thoracic Society (ATS) Guideline and the 2019 WHO guidelines both encourage the use of newer or repurposed oral agents with significant efficacy while minimizing the utility of injectable agents. Injection drugs are no longer required, and the intensive phase is no longer defined by the addition of injectable agents.

Bai et al. found that few studies are exploring the consequences of TB infection on dialysis patients [8]. In their pursuit, they found out that previous studies of TB-infected dialysis patients had inadequate treatment, more treatment failure, and 2- to 3.3-fold mortality compared to the general population. Taiwan's directly observed treatment, short-course (DOTS) policy included patients on long-term dialysis, targeting dosage adjustment of anti-TB drugs. TB incidence declined progressively along with patients receiving DOTS, showing a reduction in treatment failures, mortality, and multidrug-resistant TB rates. In contrast, two meta-analysis studies exposed that the directly observed therapy did not improve poor patient compliance with the treatment of TB.

End-stage renal failure patients are more likely to develop drug resistance due to poor patient adherence to the therapeutic regimen, adverse drug reactions, and inappropriate dosage adjustment. Multidrug resistance is uncommon in HD patients, but they are at increased risk for these reasons. There were negligible studies involving end-stage renal failure patients on HD afflicted with multidrug-resistant pulmonary TB and their treatment outcomes. Investigations regarding the relationship between these two were not yet exhausted, which could elucidate the factors resulting in poor treatment outcomes.

Therefore, the aim of this review is to assess the treatment outcome of multidrug-resistant pulmonary TB in patients with end-stage renal disease who require HD in terms of successful treatment, including cured and treatment completed, as well as the associated factors. Our secondary goal is to identify unfavorable treatment outcomes, such as treatment failure, patient death, or loss of follow-up, and the underlying factors contributing to them.

## Review

Methods

This systematic review was conducted in accordance with the Preferred Reporting Items for Systematic Reviews and Meta-Analyses (PRISMA) 2020 guidelines [9].

Eligibility criteria

Inclusion and Exclusion Criteria

Population: We included adult (more than 19 years old) chronic kidney disease (CKD) patients who needed HD and had microbiologically confirmed multidrug-resistant pulmonary TB, excluding patients who had renal allograft transplant, were on peritoneal dialysis, had extrapulmonary TB, were children, and were pregnant.

Intervention: We considered guideline (WHO)-directed treatment for multidrug-resistant pulmonary TB. We excluded studies in which intervention was related to the surgical technique and non-medical therapy.

Outcome: To be eligible for inclusion, a trial had to use a defined clinical outcome related to successful treatment (cured and treatment completed) or a poor therapeutic outcome (patient lost to follow-up, treatment failure, or patient death). Our primary outcome measure is the success rate of MDR pulmonary TB treatment among chronic renal failure patients requiring HD (cured and treatment completed). Our secondary outcome is the incidence of unfavorable treatment outcomes (patient lost to follow-up, treatment failure, or patient death).

Treatment outcome definition based on the WHO

Treatment success - the total number of treatments completed and cured [10].

Cured - a patient with pulmonary TB, bacteriologically proven, who started therapy with TB and finished treatment as advised by the national policy with evidence of bacteriological response and no failure evidence.

Treatment completed - a patient who received the recommended course of therapy in accordance with national policy but whose results did not fulfill the criteria for cured or treatment failed.

Treatment failed - a patient whose treatment plan must be stopped or switched permanently to another regimen or treatment approach.

Died - a patient who passed away either before or during treatment.

Lost to follow-up - a patient who has gone more than two months without receiving any treatment, or whose treatment was interrupted for more than two months.

We included journals published within the last 10 years (January 1, 2013, to January 1, 2023), which were in English language, had human subjects, and were peer-reviewed, in full-text, and open-access articles. Eligible studies included were randomized control trials, systematic reviews and meta-analyses, narrative reviews, case-control studies, retrospective cohort studies, prospective cohort studies, and case reports. We excluded papers published as gray literature and unpublished manuscripts.

Information sources and search strategy

From December 26, 2022, to January 15, 2023, we searched PubMed, MEDLINE, PubMed Central, ScienceDirect, Public Library of Science (PLOS), and Google Scholar using keywords such as Mycobacterium tuberculosis, pulmonary tuberculosis, multidrug-resistant pulmonary TB, chronic kidney disease, renal failure, and treatment outcome. Keywords were combined with the Boolean "AND" operator to generate results. Furthermore, the medical subject heading (MeSH) search strategy was used. The complete keyword and MeSH search strategy is shown in Table 1. We supplemented our study search by manually going through bibliographies and searching other websites, such as the WHO website.

**Table 1:** Search Strategy Using Keywords and MeSH CKD, chronic kidney disease; MDRTB, multidrug-resistant tuberculosis; MeSH, medical subject heading; PLOS, Public Library of Science; TB, tuberculosis

Search Strategy	Database Used	Number of Papers Identified	Number of Papers identified after Filter
Mycobacterium tuberculosis AND chronic kidney disease	PubMed	151	52
Multidrug-resistant pulmonary TB AND treatment outcome	PubMed	692	197
pulmonary[Title] AND tuberculosis[Title] AND chronic[Title] AND kidney[Title] AND disease[Title]	PubMed	3	3
("Tuberculosis, Multidrug-Resistant/analysis"[Majr] OR "Tuberculosis, Multidrug-Resistant/drug therapy"[Majr] OR "Tuberculosis, Multidrug-Resistant/metabolism"[Majr] OR "Tuberculosis, Multidrug-Resistant/therapy"[Majr] ) AND ( "Renal Insufficiency, Chronic/complications"[Mesh] OR "Renal Insufficiency, Chronic/drug therapy"[Mesh] OR "Renal Insufficiency, Chronic/therapy"[Mesh] )	PubMed	2	2
("Tuberculosis, Pulmonary/therapy"[Mesh] AND (( "Renal Insufficiency, Chronic/complications"[Mesh] OR "Renal Insufficiency, Chronic/drug therapy"[Mesh] OR "Renal Insufficiency, Chronic/therapy"[Mesh] )	PubMed	60	15
((((("tuberculosis, pulmonary"[MeSH Terms] OR ("tuberculosis"[All Fields] AND "pulmonary"[All Fields]) OR "pulmonary tuberculosis"[All Fields] OR ("pulmonary"[All Fields] AND "tuberculosis"[All Fields])) AND ("therapy"[Subheading] OR "therapy"[All Fields] OR "treatment"[All Fields] OR "therapeutics"[MeSH Terms] OR "therapeutics"[All Fields])) AND ("renal insufficiency, chronic"[MeSH Terms] OR ("renal"[All Fields] AND "insufficiency"[All Fields] AND "chronic"[All Fields]) OR "chronic renal insufficiency"[All Fields] OR ("chronic"[All Fields] AND "kidney"[All Fields] AND "disease"[All Fields]) OR "chronic kidney disease"[All Fields])) AND ("haemodialysis"[All Fields] OR "renal dialysis"[MeSH Terms] OR ("renal"[All Fields] AND "dialysis"[All Fields]) OR "renal dialysis"[All Fields] OR "hemodialysis"[All Fields])) AND ("drug resistance, multiple"[MeSH Terms] OR ("drug"[All Fields] AND "resistance"[All Fields] AND "multiple"[All Fields]) OR "multiple drug resistance"[All Fields] OR ("multidrug"[All Fields] AND "resistance"[All Fields]) OR "multidrug resistance"[All Fields])) AND outcome[All Fields]	PubMed Central (PMC)	924	337
Multidrug-resistant tuberculosis treatment outcome in CKD	PLOS	1584	464
Multidrug-resistant tuberculosis mdrtb AND renal failure	ScienceDirect	54	15
Multidrug-resistant pulmonary tuberculosis treatment outcome AND chronic kidney disease	Google scholar	18,100	12,800
Total		21,570	13,885

Selection process

We moved the articles to Endnote and eliminated duplicate records. The titles and abstracts of each study article were reviewed. GH (the first author) independently assessed these and used the inclusion and exclusion criteria to evaluate the full-text articles.

Quality assessment of studies

We used the Newcastle-Ottawa tool for observational studies, the Assessment of Multiple Systematic Reviews (AMSTAR) checklist for systematic reviews, and the Joanna Briggs Institute (JBI) for case reports to assess the risk of bias.

Data collection process

Primary and secondary outcomes were assessed after articles were systematically reviewed and extracted. GH extracted information independently using a standardized form. The following information was gathered: authorship and year of publication, study design, study objective, study size, population, results, treatment outcomes, and conclusion.

Results

Study Identification and Selection

**Figure 1:**
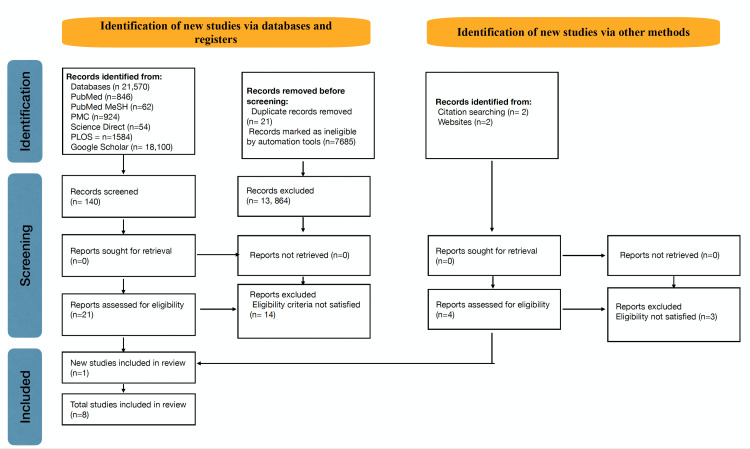
PRISMA Flowchart Displaying the Article Selection Procedure MeSH, Medical subject heading; PLOS, Public Library of Science; PMC, PubMed Central; PRISMA, Preferred Reporting Items for Systemic Reviews and Meta-Analysis

Our literature search generated 21,570 results from the aforementioned databases. After applying filters (full text, published within 10 years, age >19 years, English language, and human participants), there were 13,885 studies discovered. We removed 21 duplicate articles. We discovered 140 studies after reviewing the titles and abstracts, of which 22 were eligible for analysis. We eliminated 17 publications for failing to meet eligibility requirements when evaluated in full text. There were eight papers integrated for the final review. The full search strategy for the literature is shown using the PRISMA flow chart in Figure 1.

Outcomes Measured

The primary outcome measured was treatment success (cured and treatment completed). The secondary outcome was unfavorable treatment outcome (patient lost to follow-up, treatment failure, patient death) and associated factors.

Eight articles were evaluated for eligibility using the appropriate tools for quality assessment. Among the eight eligible studies, six observational studies (five retrospective cohort studies [2,6,8,11,12] and one case-control study [13]) were eligible for quality assessment. They were evaluated with the Newcastle-Ottawa quality assessment tool, and a 60% cutoff was used to include the study in the review. Table 2 displays the assessment of results. 

**Table 2:** Newcastle-Ottawa Quality Appraisal Tool RC, Retrospective cohort; CC, case control

Study	Study Design	Selection	Comparability	Outcome	Include/Exclude
Ali et al., 2022 [2]	RC	***	-	***	Include
Saito et al., 2019 [6]	RC	***	*	*	Include
Bai et al., 2017 [8]	RC	****	-	***	Include
Carr et al., 2022 [11]	RC	***	-	***	Include
Metry et al., 2017 [12]	RC	***	-	***	Include
Baghaei et al., 2014 [13]	CC	**	*	**	Include

The study conducted by Park et al. was the only case report that met the criteria [14]. We used the Joanna Briggs Institute (JBI) critical appraisal tool to assess the risk of bias. A cutoff of 60% was used to include the study in the analysis. The assessment results are shown in Table 3.

**Table 3:** JBI Checklist for the Case Report JBI, Joanna Briggs Institute

JBI Critical Appraisal Checklist	Park et al., 2018 [14]
Were patient’s demographic characteristics clearly described?	YES
Was the patient’s history clearly described and presented as a timeline?	YES
Was the current clinical condition of the patient on presentation clearly described?	YES
Were diagnostics tests or assessment methods and the results clearly described?	YES
Was the intervention(s) or treatment procedure(s) clearly described?	YES
Was the post-intervention clinical condition clearly described?	YES
Were adverse events (harms) or unanticipated events identified and described?	YES
Does the case report provide takeaway lessons?	YES
Include/Exclude	INCLUDE

The study by Samuels et al. was the only systematic review eligible for the quality assessment employing the AMSTAR checklist [15]. To be eligible for review, a 60% cutoff was used. The assessment results are shown in Table 4.

**Table 4:** AMSTAR Checklist for a Systematic Review AMSTAR, Assessment of Multiple Systematic Reviews Q1- Did the research questions and inclusion criteria for review include the components of PICO? Q2 - Did the report of the review contain an explicit statement that review methods were established prior to conduct of review and did the report justify any significant deviations from the protocol? Q3 - Did the review authors explain their selection of the study designs for inclusion in the review? Q4 - Did the review authors use a comprehensive literature search strategy? Q5 – Did the review authors perform study selection in duplicate? Q6 - Did the review authors perform data extraction in duplicate? Q7 - Did the review authors provide a list of excluded studies and justify the exclusions? Q8 - Did the review authors describe the included studies in adequate detail? Q9 - Did the review authors use a satisfactory technique for assessing the risk of bias (Rob) in individual studies that were included in the review? Q10 - Did the review authors report on the sources of funding for the studies included in the review? Q11 - If meta-analysis was performed, did the review authors use appropriate methods for statistical combinations of results? Q12 - If meta-analysis was performed, did the review authors assess the potential impact of RoB in individual studies on the results of the meta-analysis or other evidence synthesis? Q13 - Did the review authors account for RoB in individual studies when interpreting/discussing the results of the review? Q14 - Did the review authors provide a satisfactory explanation for and discussion of any heterogeneity observed in the results of the review? Q15 - If they performed quantitative synthesis, did the review authors carry out an adequate investigation of publication bias and discuss its likely impact on the results of the review? Q16 - Did the review authors report any potential sources of conflict of interest, including any funding they received for conducting the review?

AMSTAR Checklist	Samuels et al., 2018 [15]
Q1	YES
Q2	YES
Q3	NO
Q4	YES
Q5	YES
Q6	YES
Q7	YES
Q8	YES
Q9	YES
Q10	NO
Q11	YES
Q12	YES
Q13	YES
Q14	YES
Q15	NO
Q16	NO
Include/Exclude	INCLUDE

Study Characteristics

Eight studies were included for review, published between 2013 and 2023, with 30,062 participants. Most of the studies were retrospective cohort [2, 6, 8, 11, 12] followed by a case-control study [13], a case report [14], and a systematic review and meta-analysis [15]. Two studies reported on the therapeutic outcomes of multidrug-resistant TB in patients with chronic renal failure; one of these included HD patients. Two studies elaborated on the clinical effects and manifestations of TB; two studies explained the common adverse effects of anti-TB medications; and two studies described the risk of morbidity and mortality among renal failure patients on HD. None of the included studies were funded directly by pharmaceutical companies.

In a mixed-ethnicity renal hospital in London, United Kingdom, Ali et al. documented 143 cases of TB, with a mean age of 59 years [2]. Patients of Asian descent were found to have the highest prevalence of TB (64%), whereas those of white descent had the lowest prevalence (4%). Pneumonia and/or pleural involvement was the most common site of organ involvement, and 21 patients (14.7%) had a history of current or latent TB treatment. However, the availability and sensitivity of sputum were particularly poor, and fluid samples were often less sensitive than tissue or cellular samples. The positive likelihood ratio for interferon g release assay (IGRA) tests was 3.5 while the negative likelihood ratio was 0.45. Out of 52 cases where sensitivity testing was performed, 12 patients (23%) were found to be resistant to first-line treatment. Overall, the group had one-year and five-year survival rates of 78.5% and 60.7%, respectively, and 63 people died over the mean observation time of 48 months (from the first TB diagnosis of the research period).

Saito et al. investigated the number and nature of drug-related side effects, the number of patients whose regimens were changed due to side effects, and the TB-related hospital mortality in patients with chronic renal failure who received care with recommended dosage adjustments based on renal function [6]. Between 2005 and 2014, they studied 241 individuals who were diagnosed with pulmonary TB and were managed at Daisan Hospital at Jikei University. The average age was 64 years old, and men made up around 60% of the total. It was shown that 60 patients experienced a total of 70 adverse events (AEs), with drug-induced hepatitis being the most common and cutaneous reactions occurring in 19 patients (7.1%). In both the moderate and severe CKD groups, the incidence of AEs was greater than in the non-CKD group. While hospitalized, 14 patients (5.8%) died from TB, and the overall sputum culture conversion rate at two months was 78%.

In a retrospective cohort study, Bai et al. investigated the clinical impact of pulmonary TB in dialysis patients between 1999 and 2013 using the Taiwan National Health Insurance Research Database [8]. The total sample size was 9,965 individuals, including 1,993 TB patients and 7,972 non-TB patients. Based on DOTS implementation, the TB patients in 1993 were split into pre-DOTS (n = 1,069) and DOTS (n = 924) subgroups. When compared to non-TB patients, pre-DOTS TB patients had a significantly higher occurrence of illnesses such as hospitalization, pneumonia, and an intensive care unit (ICU) stay of more than seven days, but DOTS TB patients did not. Patients with TB in the entire sample had significantly higher risks of requiring inotropic drugs, ventilator support for more than 21 days, and death compared to non-TB patients. The incorporation of DOTS strategy had no effect on the risks. Within two years of initiating TB treatment, the mortality disparity between TB and non-TB patients decreased steadily.

To assess the link between CKD and all-cause mortality in a setting with a low TB prevalence, Carr et al. managed a retrospective cohort analysis with 653 individuals from a huge Australian healthcare system that draws its information from a national database of recorded TB infections between 2010 and 2018 [11]. High levels of correlation were established between age, diabetes, renal function, and total mortality. The hazard ratio for all-cause mortality was 22.1 for the estimated glomerular filtration rate (eGFR) between 30 and 44 ml/min and 34 for eGFR < 30 ml/min. This rose dramatically with decreasing eGFR values. Increased overall mortality has been attributed to the presence of pulmonary and extra-pulmonary site involvement at the same time. Of the 520 cases for which culture-based diagnosis was available, 23 died of TB; of these, 22 (or 95.7%) had fully sensitive TB, 1 (or 4.3%) had TB resistant to isoniazid, and none had TB resistant to multiple drugs.

Metry et al. examined the prognosis, therapy, drug resistance, mortality, and morbidity of patients with chronic renal disease [12]. In a retrospective analysis of all TB cases treated at the Royal Hospital between 2006 and 2015, 581 patients with active TB were included, 37 of whom had renal dysfunction. The most prevalent manifestations were pulmonary TB (64.9%) and TB lymphadenopathy (10.8%). The CKD group had more positive skin tests than the HD and renal transplant groups. TB cultures were among the most positive tests in the renal dysfunction group, whereas bronchoalveolar lavage (BAL) and skin tests were the least positive. Thirty-one (83.8%) of the renal dysfunction patients completed their treatment, six (16.2%) failed treatment, and three expired.

Baghaei et al. conducted a retrospective cohort analysis on adults with TB and chronic renal failure (CRF) to determine the success of TB treatment [13]. There were 220 cases of TB (55 with CRF and 165 without), 32.7% were on HD, 90.9% had pulmonary TB, and 43.6% were male. The median interval between chronic renal failure (CRF) and TB diagnosis was seven months. Forty patients developed drug-induced hepatitis (DIH), 15 (27.3%) of whom had CRF and 25 (15.2%) did not. The correlation between CRF and DIH was not seen after controlling for demographic factors such as age, gender, and the presence of viral hepatitis. Notably, the number of patients who self-reported other adverse medication reactions, such as dermatitis and neuropathy, was comparable across those with and without CRF (23% vs. 39%, P = 0.57). Eighteen patients were lost to follow-up prior to therapy completion; four (7.3%) had CRF and fourteen (8.5%) did not. Of the remaining 202 patients, there were 15 fatalities and no treatment failures. At six months, the cumulative incidence of death from all causes was greater in individuals with CRF compared to those without.

Park et al. discussed two case reports of male ESRD patients on HD from South Korea who were diagnosed with pulmonary MDR-TB and given bedaquiline-containing regimens to treat their condition [14]. The average age of these patients was 49.5 years, and they suffered from hypertension, diabetes, and hepatitis C infection. In these patients, they studied the safety of bedaquiline and how well it was tolerated. Both patients experienced favorable outcomes from treatment, as evidenced by the absence of significant cardiac events or significant QT prolongation.

Samuels et al. conducted a systematic review and meta-analysis study of 18 studies and 257 participants with MDR or XDR-TB and included 48 studies published from 1996 to 2016 with a median population of 235 (60-1768) [15]. Their primary goal was to investigate the relationship between comorbidities and the risk of MDR-XDR-TB treatment failure. People living with HIV and alcoholics had a higher relative risk of treatment failure overall. Patients with CKD lacked sufficient data to examine the major outcome measure for poor treatment outcomes (failure, mortality, and default).

Table 5 contains a summary of the characteristics of the eight included studies, along with the authors, the year the studies were published, the study designs, the objectives of the studies, the sample sizes, the populations studied, the results, the outcomes of treatment, and the conclusions.

**Table 5:** Summary and Characteristics of Included Studies AE, adverse events; BAL, bronchoalveolar lavage; CC, case control; CKD, chronic kidney disease; CR, case report; CRF, Chronic renal failure; DIH, Drug-induced hepatitis; DOTS, Directly observed treatment, short-course; eGFR, estimated glomerular filtration rate; ESRD, end-stage renal failure; HD, hemodialysis; ICU, Intensive Care Unit; KTX, kidney transplant; MDR, Multidrug resistant tuberculosis; MDR PTB, multidrug-resistant pulmonary tuberculosis; PTB, pulmonary tuberculosis; RC, retrospective cohort; SR & MA, systematic review and meta-analysis; TB, tuberculosis; XDR TB, extensively drug-resistant tuberculosis

Author/Year of Publication	Study Design	Objective	Sample Size	Study Population	Results	Treatment Outcome	Conclusion
Ali et al., 2022 [2]	RC	To illustrate how TB manifests in dialysis and kidney transplant recipients, using various diagnostic methods.	143	82% (118) on HD; 60.1% (86) PTB	TB was most common in Asian patients, with prior treatment present in 21 cases. Sputum and pleural fluid had low sensitivity. Drug sensitivity testing was available in 52 cases, with 12 patients showing resistance to a first-line drug.	45% (63) deaths from TB diagnosis; 23% drug resistance;1-year survival (78.5%), 5-year survival (60.7%) from the time of diagnosis	TB reactivation is common in patients with kidney failure, but diagnostic sensitivity is low and treatment is often initiated without confirmation despite high rates of drug resistance.
Saito et al., 2019 [6]	RC	To investigate drug-related side effects and Tb-related mortality in CKD patients.	241	Active pulmonary TB;7.5% (18) severe CKD;11 on dialysis	AEs were higher in moderate and severe CKD than non-CKD group, with 14 patients dying from tuberculosis and 78% sputum culture conversion rate.	70 side effects observed in 60 patients;11.6 % (28) drug - induced hepatitis; 7.9% (19) cutaneous reactions; 5.8% (14) death due to TB during hospitalizations	CKD patients can use dose adjustments based on renal function for the prevention of TB-related death.
Bai et al., 2017 [8]	RC	To investigate the clinical effects of pulmonary TB among long-term dialysis patients	9,965	TB patients (n=1993) vs no TB (n=7972); 98.2% (1958) on HD	TB infected patients had higher mortality risk, but DOTS implementation decreased morbidities such as pneumonia, hospitalization, and ICU stay.	Patients who received anti-TB medications for 180-224 days experienced a reduction in relapse.	DOTS reduces morbidity and TB relapse rates in dialysis patients with PTB.
Carr et al., 2022 [11]	RC	To evaluate CKD and total mortality in regions with low TB prevalence	653	1.5% (8) MDR TB; 2.9 % (18) eGFR <30ml/min	All-cause mortality was greater in chronic renal disease patients with starting eGFR level of 45ml/min than TB cases with eGFR >60ml/min.	(3.8%) 25 people died overall	People with renal impairment have an increased risk of dying from all causes in low TB prevalence.
Metry et al., 2017 [12]	RC	To assess renal disease prognosis, treatment, drug resistance, mortality, and morbidity.	581	19 CKD (5 HD, 13 KTX); PTB 296 (35.24%)	PTB (64.9%) and TB lymphadenopathy (10.8%) were the most common presentations in CKD and kidney disorder groups. TB cultures were the most positive tests, while BAL and skin tests were the least positive.	84% (31)w/ CKD completed course of treatment; 8% (3) lost to follow-up; 8.1% (3) mortality; 0.4% (1) developed MDR TB	Improvements in registers and screening are needed to capture and detect TB in kidney dysfunction.
Baghaei et al., 2014 [13]	CC	To ascertain the effect of chronic renal failure on the success of TB treatment.	220	55 CRF vs 165 no CRF; 32.7% (18) on HD; 90.9% (200) PTB	CRF was no longer associated with DIH after controlling demographics, and self-reported adverse drug reactions were comparable between patients with and without CRF.	40 (18.2%) patients developed DIH; no treatment failure; 15 (6.8%) died during anti-TB treatment	TB infected chronic renal failure patients are more likely to die.
Park et al., 2018 [14]	CR	To discuss two ESRD patients who received bedaquiline treatment for pulmonary MDR-TB	2	MDR PTB ESRD on hemodialysis	Bedaquiline-containing regimens had successful treatment outcomes and no significant cardiac events or QT prolongation.		Research is needed to increase effectiveness of bedaquiline and manage drug-resistant TB in patients with ESRD.
Samuels et al., 2018 [15]	SR & MA	Primary objective: to determine to determine the association between co-morbidities and treatment failure for MDR/XDRTB. Secondary objective: to determine the relationship between each comorbid disorder and treatment outcome.	18, 257	At least 50 participants with MDRTB and/or XDRTB that has been microbiologically confirmed.	48 included studies published from 1996 to 2016 with a median population of 235 (60-1768).	For CKD outcomes, only two studies with participants w/ MDR/XDR and CKD; due to lack of data, primary and secondary outcomes could not be analyzed.	Improved reporting of comorbid conditions can help identify successful treatment outcomes for various subpopulations.

Discussion

Juno and colleagues conducted exhaustive research on the relationship between end-stage renal disease and increased susceptibility to infectious diseases like TB [4]. Mucosal-associated invariant T cells (MAIT) can differentiate vitamin B metabolites formed by a variety of bacteria, including Mycobacterium tuberculosis (MTb), which plays an important role in TB prevention in the lung. In contrast, patients with active TB who had renal failure showed a decrease in peripheral MAIT cells and a rise in their number in the lungs, where they are activated and recruited during lung infection. The reduction of peripheral blood MAIT cells, as well as alterations in the expression of tissue homing receptors and Granulocyte-Macrophage colony stimulating Factor (GM-CSF) synthesis in the lungs, may help to create an immune environment favorable for bacterial replication. While CXC motif chemokine receptor 3 (CXCR3) expression was reduced in the MAIT population, CC motif chemokine receptor 6 (CCR6) and CXC chemokine receptor type 6 (CXCR6) expression was increased in ESRD patients, potentially leading to differences in cell trafficking to tissues. As a result, changes in the MAIT phenotype in ESRD patients could indicate increased recruitment to damaged tissue and disappearance from circulation. Although interferon-g (IFN- g) production can be maintained in ESRD patients by stimulating MAIT cells with IL-12 or IL-18, low levels of tumor necrosis factor (TNF) production in response to microbial activation suggest that the response to bacterial infection is dysfunctional. TNF inhibitors have been linked to TB reactivation, emphasizing the role of TNF in MTb control. The reduction in CXCR3 expression, the possibility of MAIT cells homing to inflamed kidney tissues, and the shift in cytokine production away from IFN/TNF and toward GM-CSF all likely contribute to an immune environment more receptive to MTb infection and reactivation.

**Figure 2:**
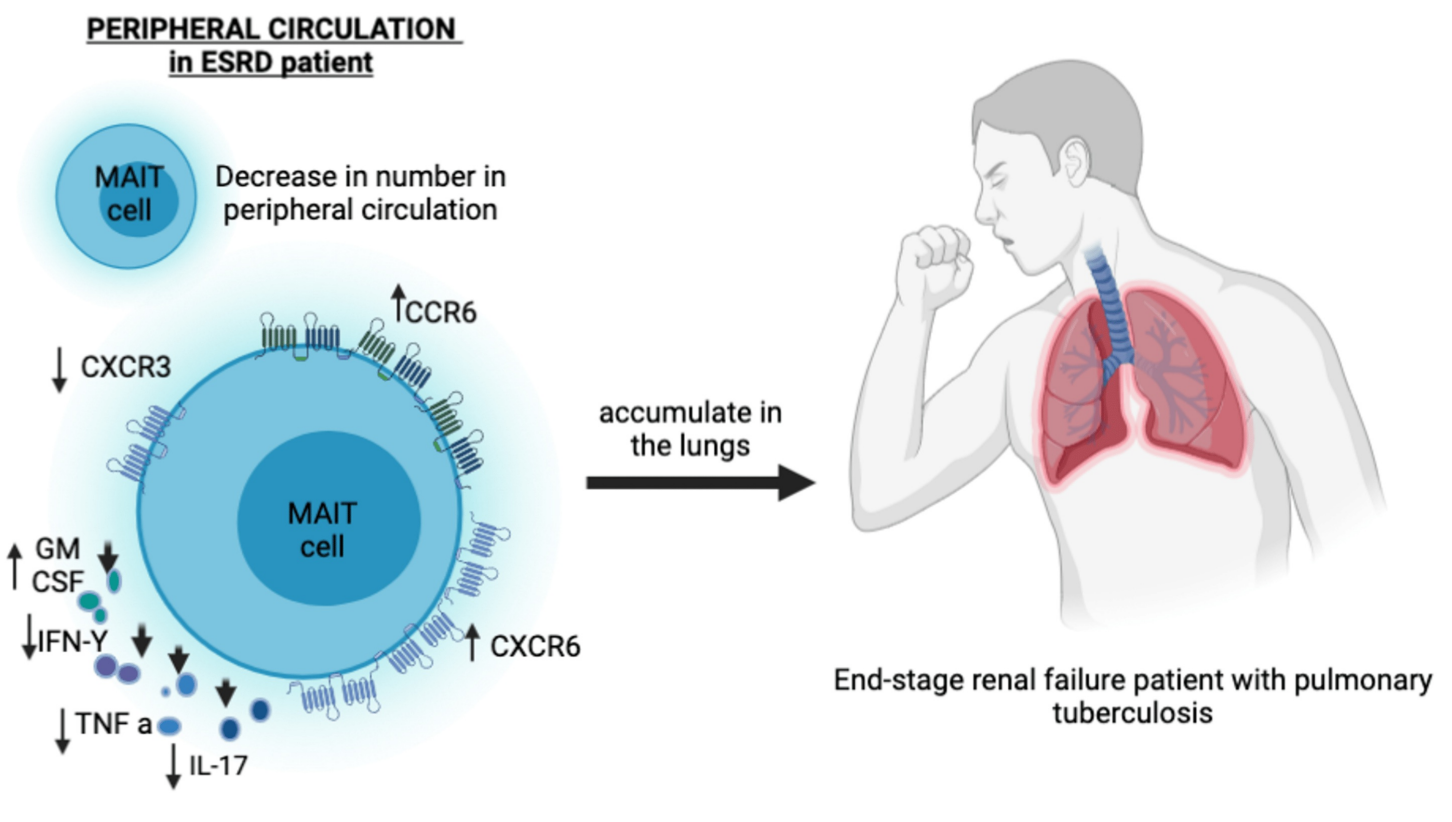
Mucosal-Associated Invariant T-Cell Depletion and Altered Chemokine Receptor Expression During Mycobacterium Tuberculosis Infection in an End-Stage Renal Failure Patient CXCR3, CXC motif chemokine receptor 3; CCR6, CC motif chemokine receptor 6; CXCR6, CXC chemokine receptor type 6; ESRD, end-stage renal disease; GM-CSF, granulocyte-macrophage–colony-stimulating factor; IFN g, interferon g; IL- 17, interleukin-17; MAIT, mucosal-associated invariant T-cell; TNF a, tumor necrosis factor a Figure created by the corresponding author GH.

Furthermore, T cell populations such as gamma delta (gd) T-cells are thought to protect against Mycobacterium TB infection and can boost CD4+ and CD8+ T cell responses [3]. T-cells from ESRD patients with latent TB infection significantly disrupt their function, resulting in TB latency. Figure 2 shows the reduction of MAIT cells in the circulation and altered chemokine receptor expression during TB infection. 

MDR-TB and XDR-TB are major impediments to global TB elimination [12]. Multidrug-resistant TB is described as resistance to rifampicin and isoniazid, necessitating treatment with second-line drugs [1]. Since the 1990s, the WHO has assessed data on the efficacy of various drug formulations and durations of treatment [16]. In 2016, patients with MDR/RR-TB strains that are not resistant to fluoroquinolones or second-line injectable agents were recommended a standardized shorter treatment regimen (9-12 months), while longer regimens (18-20 months) remained an option for patients who did not qualify for the shorter option. The WHO assessed new evidence, resulting in revised recommendations that balanced the effectiveness and risks of new regimens or changes to recommended regimens. Several initiatives have been launched recently to reduce treatment duration, with shorter regimens achieving a cure with no relapse in 80% of cases or more.

MDR TB Treatment Outcome in CKD

It is uncommon for end-stage renal failure patients to develop multidrug-resistant TB, and negligible studies are probing their association. From the facts gathered, we believe this is the first systematic review investigating the relationship between multidrug resistance in pulmonary TB and its therapeutic outcomes among patients with renal dysfunction requiring HD. There were two studies that assessed this relationship. Park et al. described two case reports of a successful therapeutic outcome for HD patients with multidrug-resistant pulmonary TB using a regimen containing bedaquiline [14]. However, additional studies were needed to determine its safety and efficacy among end-stage renal failure patients. Samuels et al. conducted the only systematic review and meta-analysis examining the relationship between multidrug-resistant TB, specific comorbidities, and therapy outcomes [15]. Their study revealed that people living with HIV (PLWH), diabetic patients, smokers, and individuals with alcohol misuse had a significant risk for unsuccessful treatment outcomes, mortality, and treatment failure for MDR-TB. Their study, however, revealed that the primary outcome measure for unfavorable treatment outcomes (failure, death, and default) could not be analyzed due to a lack of data among patients with CKD.

Novel Drug for MDR TB

In a case report by Park et al., treatment outcomes for a bedaquiline-containing regimen for two HD patients with multidrug-resistant TB were successful, without clinically significant cardiac events or significant QT prolongation [14]. In end-stage renal failure patients, QT prolongation is frequently observed and may be an independent predictor of mortality. As a result, including bedaquiline in an MDR-TB regimen in patients with end-stage renal failure may raise safety concerns. Bedaquiline is primarily eliminated in the feces, and its excretion in the urine with its M2 metabolite is negligible. Nonetheless, the influence of dialysis on Bedaquiline pharmacokinetics is unknown. The pharmacokinetics of drugs that are not eliminated by the kidneys may be affected by renal impairment due to changes in drug-metabolizing enzymes or drug transporters. Thus, therapeutic drug monitoring (TDM) may benefit these patients. In addition, there are no definite guidelines for TDM available for end-stage renal failure patients. To better support the use of bedaquiline as a treatment for patients with MDR-TB and ESRD, more evidence is required.

Successful Therapeutic Outcomes of PTB in CKD

In a case report, Park et al. reported the favorable treatment outcome of two HD patients who developed multidrug-resistant pulmonary TB [14]. The first patient was started on a combination of pyrazinamide, moxifloxacin, cycloserine, amikacin, linezolid, and bedaquiline (200 mg thrice weekly, continued for 24 weeks) with a total duration of treatment of 607 days [11]. Amikacin and linezolid were permanently discontinued due to adverse effects such as peripheral neuropathy and hearing disturbances. The other patient with CKD, who subsequently required HD, was initiated on a combination of pyrazinamide, levofloxacin, amikacin, prothionamide, and cycloserine. Amikacin was permanently discontinued due to its nephrotoxic side effects. Bedaquiline was subsequently added after two months. The treatment duration lasted for 653 days. Both patients had no clinically significant cardiac events during treatment.

A satisfactory treatment outcome was found in the research carried out by Metry and colleagues, as 84% of the patients diagnosed with renal failure were able to finish the prescribed course of anti-TB treatment [12]. Due to frequent and routine follow-ups in addition to improved access to medical treatment, the average length of symptoms was shorter in the group of people with renal dysfunction (14 days, compared to 33.4 days in the general population) before a diagnosis of TB.

Treatment success is essential for the management of TB since the failure of treatment encourages the spread of active TB and the development of resistance to anti-TB drugs [6]. Nonetheless, an individual's death may arise from a poor treatment response or a failure of therapeutic interventions. Comorbidity is a major contributor to therapeutic failure, as it increases the complexity of care, the risk of treatment failure, and, ultimately, death. Depending on the delay in diagnosis and whether therapy was discontinued due to noncompliance or adverse drug reactions, the overall mortality of TB patients with uremia ranges from 16.4% to 36.8% across studies.

Adverse Effects of Anti-TB therapy in ESRD Patients

Two studies reported several adverse effects of anti-TB medications in end-stage renal failure patients. ESRD patients treated with anti-TB drugs had a higher risk of developing drug-induced hepatitis and death while receiving treatment [13]. According to Baghaei et al., numerous studies have discovered an unusually high prevalence of hepatic side effects linked to anti-TB medication in dialysis patients. The most common side effect was drug-induced hepatitis (11.6%), followed by cutaneous reactions (7.9%) [6]. In this study, renal failure patients experienced more adverse effects even after renal function-based dose modification than non-CKD patients. A pharmacokinetic investigation found that hepatic acetylation caused substantial isoniazid (INH) build-up in CKD patients compared to normal subjects. Therefore, INH accumulation persisted even after the recovery of symptoms and laboratory findings.

Physicians may briefly discontinue anti-TB medication depending on the severity of the reaction, and permanent discontinuation is done when undesirable effects recur [6]. It is possible that a longer amount of time from the regimen will improve the efficacy of desensitization therapy, thereby lowering the risk of developing resistance to second-line drugs. A study showed that dose modification based on renal function for patients with CKD results in positive outcomes in terms of therapeutic effectiveness. 

**Figure 3:**
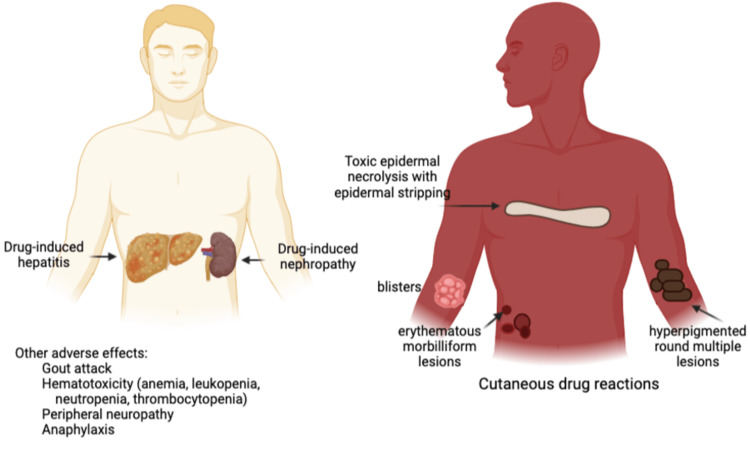
Common Adverse Drug Reactions of Anti-tuberculosis Medications Figure created by the corresponding author GH.

Figure 3 shows the common adverse drug reactions of anti-TB medications. Drug-induced hepatitis is the leading adverse effect followed by cutaneous reactions described as toxic epidermal necrolysis with epidermal stripping, blisters, erythematous morbilliform lesions and hyperpigmented round multiple lesions [6,13,17]. Other adverse effects are drug-induced nephropathy, gouty arthritis, hematotoxicity, peripheral neuropathy, and anaphylaxis [6,13].

Risk Factors for Morbidity and Mortality

Baghaei and Carr et al. found that patients who had chronic renal disease beginning at an eGFR of 45 ml/min had a higher risk of dying from any cause compared to TB patients whose eGFR was greater than 60 ml/min [11,13]. They are also at a higher risk of morbidities such as pneumonia, hospitalizations, and ICU stays of more than seven days [8]. In the study that was conducted by Bai and colleagues, the use of DOTS did not reduce the risks of severe morbidities involving inotropic agents, ventilator use, or mortality among TB non-TB patients. The most likely explanations are cardiovascular disease and sepsis which are the two most common causes of death in patients on long-term HD. In TB-infected dialysis patients, the coexistence of TB and other common diseases, such as diabetes mellitus, coronary artery disease (CAD), and heart failure (HF), can aggravate the medical condition and lead to serious morbidity. After starting TB medication, the risk of death decreased steadily until it approached the risk seen in non-TB patients after two years. Meanwhile, the similar rates of TB recurrence among patients who received anti-TB medications for 180-224 days and 225 days suggested that adequate and shorter combination therapy could result in effective TB treatment. DOTS implementation under the supervision of community health workers and nephrologists may overcome poor compliance, the most significant barrier to effective TB treatment.

Ali et al. reinforced the concept that HD patients with pulmonary TB had a lower chance of survival due to a failure to detect drug resistance [2]. Clinical disease in dialysis patients frequently began soon after dialysis initiation, with 66 (54%) diagnoses occurring within the first year. While active TB may cause or hasten renal disease, TB reactivation as part of an immune reconstitution syndrome following dialysis induction is a more likely explanation. With increased invasive diagnostic sampling (microbiologically or histologically), the diagnosis was established in 87 patients (61%), resulting in a rise in the proportion of confirmed versus non-confirmed TB over time. Sputum (18%) and pleural fluid (12%) had lower sensitivity rates compared to tissue samples such as bronchoscopic lymph node aspiration (75%) and other lymph node sampling (92%). In the 128 (90%) cases where it was known, the median disease duration at the time of diagnosis was 2.0 (1.0-3.5) months, but it tended to be longer in those with unconfirmed TB. From the time of diagnosis, these patients with pulmonary TB had a 78.5 percent one-year survival rate and a 60.7 percent five-year survival rate.

Carr and colleagues highlighted the role of severe renal dysfunction (eGFR<30ml/min) in TB-related mortality, which may be attributable to the additive effects of malnutrition and immunosuppression [11]. They considered the possibility of anti-TB medication selection or dose adjustment to TB disease undertreatment, contributing to TB-related death. They advocated for pharmacokinetic studies to assess undertreatment and dose optimization. In addition, they recommended accurate TB screening and diagnostic testing, as well as the safety and efficacy of treatment for patients with renal failure.

Limitations

Our study was limited to a small sample of HD patients with multidrug-resistant pulmonary tuberculosis. As a result, the primary and secondary outcomes of interest were not thoroughly investigated.

## Conclusions

Patients with CKD, particularly those on dialysis, are extremely susceptible to tuberculosis due to the immunosuppression caused by multiple factors. This infection poses a threat of morbidity and mortality to the population. Controlling comorbidities, ensuring early tuberculosis detection and treatment, detecting drug resistance, and ensuring DOTS adherence can reduce these risks. All new dialysis patients should routinely undergo IGRA testing. In this population, drug-induced hepatitis and cutaneous reactions are the most common adverse drug reactions associated with anti-tuberculosis medication. Due to their altered drug metabolism, these patients require a therapeutic drug monitoring guideline in order to reduce adverse effects and even mortality. Additional research is necessary to determine the safety, efficacy, and outcomes of therapeutic regimens in this population with multidrug-resistant tuberculosis.

## References

[1] (202220222022). Global Tuberculosis Report 2022. https://www.who.int/teams/global-tuberculosis-programme/tb-reports/global-tuberculosis-report-2022.

[2] Ali M, Dosani D, Corbett R (2022). Diagnosis of tuberculosis in dialysis and kidney transplant patients. Hemodial Int.

[3] Juno JA, Waruk JL, Harris A (2017). γδ T-cell function is inhibited in end-stage renal disease and impacted by latent tuberculosis infection. Kidney Int.

[4] Juno JA, Waruk JL, Wragg KM (2018). Mucosal-associated invariant T cells are depleted and exhibit altered chemokine receptor expression and elevated granulocyte macrophage-colony stimulating factor production during end-stage renal disease. Front Immunol.

[5] Lu M, Sue YM, Hsu HL (2022). Tuberculosis treatment delay and nosocomial exposure remain important risks for patients undergoing regular hemodialysis. J Microbiol Immunol Infect.

[6] Saito N, Yoshii Y, Kaneko Y (2019). Impact of renal function-based anti-tuberculosis drug dosage adjustment on efficacy and safety outcomes in pulmonary tuberculosis complicated with chronic kidney disease. BMC Infect Dis.

[7] Nahid P, Mase SR, Migliori GB (2019). Treatment of drug-resistant tuberculosis. An Official ATS/CDC/ERS/IDSA Clinical Practice Guideline. Am J Respir Crit Care Med.

[8] Bai KJ, Huang KC, Lee CH, Tang CH, Yu MC, Sue YM (2017). Effect of pulmonary tuberculosis on clinical outcomes of long-term dialysis patients: Pre- and post-DOTS implementation in Taiwan. Respirology.

[9] Page MJ, McKenzie JE, Bossuyt PM (2021). The PRISMA 2020 statement: an updated guideline for reporting systematic reviews. BMJ.

[10] (202020213030). Meeting report of the WHO expert consultation on drug-resistant tuberculosis treatment outcome definitions, 17-19 November. https://www.who.int/publications/i/item/9789240022195.

[11] Carr BZ, Briganti EM, Musemburi J, Jenkin GA, Denholm JT (2022). Effect of chronic kidney disease on all-cause mortality in tuberculosis disease: an Australian cohort study. BMC Infect Dis.

[12] Metry AM, Al Salmi I, Al-Abri S, Al Ismaili F, Al Mahrouqi Y, Hola A, Shaheen FA (2017). Epidemiology and outcome of tuberculosis in immunocompromised patients. Saudi J Kidney Dis Transpl.

[13] Baghaei P, Marjani M, Tabarsi P (2014). Impact of chronic renal failure on anti-tuberculosis treatment outcomes. Int J Tuberc Lung Dis.

[14] Park S, Lee KM, Kim I, Mok J (2018). The use of bedaquiline to treat patients with multidrug-resistant tuberculosis and end-stage renal disease: a case report. Int J Infect Dis.

[15] Samuels JP, Sood A, Campbell JR, Ahmad Khan F, Johnston JC (2018). Comorbidities and treatment outcomes in multidrug resistant tuberculosis: a systematic review and meta-analysis. Sci Rep.

[16] WHO consolidated guidelines on tuberculosis. Module 4: treatment - drug-resistant tuberculosis treatment, 2022 update. Geneva: World Health Organization; 2022. License: CC BY-NC-SA 3.0 IGO. https://www.who.int/publications/i/item/9789240063129.

[17] Lehloenya RJ, Dheda K (2012). Cutaneous adverse drug reactions to anti-tuberculosis drugs: state of the art and into the future. Expert Rev Anti Infect Ther.

