# Correction: Unraveling the Secrets Behind the Multidrug-Resistant Tuberculosis Treatment Outcome in Chronic Renal Failure Patients Requiring Hemodialysis: A Systematic Review

**DOI:** 10.7759/cureus.c320

**Published:** 2025-09-25

**Authors:** Grethel N Hernandez, Kofi Seffah, Mustafa Abrar Zaman, Nimra Awais, Travis Satnarine, Ayesha Haq, Deepkumar Patel, Sai Dheeraj Gutlapalli, Areeg Ahmed, Safeera Khan

**Affiliations:** 1 Internal Medicine, California Institute of Behavioral Neurosciences & Psychology, Fairfield, USA; 2 Internal Medicine, Piedmont Athens Regional, Athens, USA; 3 Internal Medicine, St. George's University School of Medicine, Newcastle upon Tyne, GBR; 4 Research, California Institute of Behavioral Neurosciences & Psychology, Fairfield, USA; 5 Pediatrics, California Institute of Behavioral Neurosciences & Psychology, Fairfield, USA; 6 Neurology, California Institute of Behavioral Neurosciences & Psychology, Fairfield, USA; 7 Internal Medicine, Richmond University Medical Center, Staten Island, USA; 8 Internal Medicine Clinical Research, California Institute of Behavioral Neurosciences & Psychology, Fairfield, USA

This article has been corrected to fix the following typo in the PRISMA diagram: "Articles excluded" has been corrected from 13,725 to 13,864.

